# A New Housing Mode in a Regional Landscape of Care: A Sociocultural Psychological Study of a Boundary Object

**DOI:** 10.1007/s42087-023-00363-5

**Published:** 2023-09-20

**Authors:** Fabienne Gfeller, Tania Zittoun

**Affiliations:** Faculty of Humanities, Institute of Psychology and Education, University of Neuchatel, Espace Tilo-Frey 1, 2000 Neuchatel, Switzerland

**Keywords:** Housing, Boundary object, Landscapes of care, Regional case study, Sociocultural psychology, Ageing

## Abstract

The study of ageing, which received growing attention over the past 30 years, has progressively realised the importance of the cultural, historical, and socio-economical environment for the various courses of ageing. However, we believe that it could be further conceptualised. First, we propose to enrich it through the notion of “landscape of care” developed by geography. Second, the distinction developed by sociocultural psychologists between sociogenesis, microgenesis, and ontogenesis is useful to articulate different scales of the landscape of care and to consider individual trajectories. Finally, the notion of boundary object leads us to discuss how a specific object might play a bridging function in this landscape. We draw on a regional case study carried out in a Swiss canton where the building of “flats with referees” is part of a new policy that aims at adapting the care and support network to demographic change and to favour ageing in place. Our hypothesis is that these flats may have a function of boundary object as they lead various actors to collaborate. Based on observations, desk research, and interviews, the study shows that on a sociogenetic level, these flats have a bridging function. However, on ontogenetic and microgenetic levels, divergences and misunderstandings hinder these flats to fully achieve this function. By examining the changes in the landscape of care, this article contributes to a better understanding of people’s trajectories within their sociocultural environments.

## Introduction

HomAge^1^ is a research project in which we examine the transformations of housing modes in a Swiss Canton, and their consequences for the development of older persons. This study aims at contributing both to psychology of ageing and to a local dialogue with authorities in charge of the implementation of policies regarding demographic ageing. It is constructed as a four years regional case study. The Canton we study is a French-speaking region of less than 200,000 inhabitants, with a few urban centres and a population otherwise spread in rural areas (villages or isolated houses) in a diversified landscape (lakes and rivers, mountains and valleys, fields and forests). Currently, about 20% of people are more than 65 years old (retirement age). Since 2012, this Canton implemented a new policy to reduce the institutionalisation of older persons, and promoted a model of “ageing in place” (Gfeller et al., 2021). One of the innovations promoted by this new policy consists of a new type of housing, flats with referees (in French, “appartements avec encadrement”): non-medicalised, these flats are supposed to support older persons’ autonomy, quality of life, and social relations. As psychologists, we are interested in the way this changing environment might support older people’s development. The study of ageing has been gaining growing attention in social sciences, medical sciences, and psychology over the past 30 years, due to an international awareness of increased life expectancy — although this may stagnate or decrease in some groups and some countries (Gutin & Hummer, 2021; Kohli, 2017; Kohli et al., 2020) — and the general decline of natality in most of the countries of the world (Population Division, 2020).

In psychology, lifespan approaches introduced a life course perspective in the study of the old and very old (Baltes & Lang, 1997; Baltes & Mayer, 2001; Levy et al., 2005). Approaches have been long focused either on cognitive functioning (Baltes et al., 1999), searching for the emergence of an aged-related wisdom (Staudinger & Glück, 2011), or on more encompassing identity development or experiencing (Erikson & Erikson, 1998; Gubrium, 2011; Kenyon et al., 2011). Several grounding researches have been produced in social gerontology and led to the development of different psychosocial theories of ageing, notably disengagement theory (Cumming & Henry, 1961) and activity theory (Havighurst & Albrecht, 1953), and more recently continuity theory (Atchley, 1989) and socioemotional selection theory (Carstensen, 1993). Current approaches tend to focus on neurological or biological aspects (Thomas & Gutchess, 2020) or are more integrative and question the approach associating ageing with pathology (Bengston et al., 2012; Schaie, 2016) and assistive technologies (Tournier, 2022).

To fight against a general societal negative representation of ageing, usually associated with the idea that ageing consists mainly in decline, research has proposed more nuanced ways to consider the development of older persons by introducing notions such as normal, successful, pathological, positive, or meaningful ageing (Gergen & Gergen, 2001; Lieblich, 2014; Schaie, 2016).

Research into ageing has also progressively realised the importance of the cultural, historical, and socio-economical context for the various courses of ageing. Among psychosocial or ecological approaches, sociocultural psychology assumes that the social, cultural, and material environment is deeply related to the person’s development. This relation between the person and their environment is considered as mediated by semiotic means and tools, and meaning making processes as well as people’s activities are central units of analysis (Rosa & Valsiner, 2018; Valsiner, 2002; Valsiner & Rosa, 2007). It has recently started to study the development of older people (Grossen et al., 2021; Zittoun & Baucal, 2021), assuming, as other developmental approaches also do, that they do learn and develop, whatever their chronological age or their official retirement status and, more specifically, that they do so in occasions of ruptures that trigger a reconfiguration or transformation of people’s spheres of experience(Zittoun et al., 2021). However, in the present state-of-the-art, sociocultural psychology has also its shortcomings. One of them is that it lacks to fully account of what exactly the term “environment” covers. While it is commonly understood in a relational way — as a subjective, experienced environment — what this implies, notably in terms of methodology and analysis, variates among approaches, authors, or even different works of a same author. For instance, in Vygotsky’s text “the problem of the environment” (1934/1994), where the environment is defined in a relational way as ever changing depending on the child’s development, the example focuses on a family, the behaviours of its members, and the relations that constitute it, and thus on human beings. In Valsiner’s work, environment is analysed mostly in semiotic terms (see, e.g., Valsiner, 2014), while, drawing on James Gibson’s work, other authors see it as offering affordances (e.g. Glăveanu, 2012). When considering the environment relevant to understand human activity or human development and learning, researchers might include material characteristic of objects (Tanggaard, 2013), institutions (Daniels, 2012), historical changes such as revolutions (Zittoun, 2019), or animals (Gfeller, 2019), among many other examples. In this paper, we borrow the notion of “landscape of care” proposed in human geography (Milligan & Wiles, 2010), which provides a relevant analytical tool to approach our fieldwork. We then complement it through our sociocultural approach, and with the notion of boundary object.

In the first part of this article, we introduce the theoretical framework which guided our study. In the next section, we present the research project HomAge, on which we draw for our analysis. In the third section, we report the results of our analysis of the change of landscape of care introduced by flats with referees and show that these flats are liable to have a function of boundary object between the various actors of this landscape, which we discuss in a final section.

## Theoretical Framework

Sociocultural psychology offers an important route to address the development of individuals in complex societies, yet is still looking for efficient ways to precisely articulate people’s lived experiences, with the constrained and opportunities set by their institutional and natural environments. Other social sciences can help us to do so, one being human geography.

### Landscapes of Care: a Sidestep in Human Geography

Among the existing theorisations of the environment and its role in the ageing process, the notion of “landscape of care” is especially interesting. In the social sciences, the notion of care (Gilligan, 1982) acknowledges the interdependencies and vulnerabilities of every human being and aims at not considering these as essentially negative (Laugier, 2012). It is particularly relevant for studying ageing as, while older people are not necessarily more vulnerable or in need of more care than younger people, and their experience cannot be reduced to vulnerability, risk and care, older people as a statistical category present an increased need for care (Andreani & Marquis, 2023). The notion of landscape of care has usefully expanded the notion of care beyond a relationship between care provider and care receiver to encompass everything that may support the lives of people in vulnerabilising situations, as it may be the case for certain older people. “Landscapes of care” have been conceptualised as “the provision of practical and emotional support” (Milligan & Wiles, 2010, p. 737) and have two main features: the interrelatedness of care and its spatiality. On the one hand, recognising its interrelatedness leads to examine the “complex network of actors and actions involving multidirectional flows and connections” (Milligan & Wiles, 2010, p. 737). On the other hand, the notion of “landscapes of care” focuses on the spatial dimension of care. It includes physical and social spaces so as to theorise “how particular attributes of the physical and social environment, and the meanings associated with place identity can have a profound impact on health and well-being, and on care and caregiving” (Skinner & Herron, 2020, p. 57). The scales of these spaces range from international dynamics to local, interpersonal relations: “Such landscapes can encompass the institutional, the domestic, the familial, the community, the public, the voluntary and the private as well as transitions within and between them” (Milligan & Wiles, 2010, p. 738).

Researchers working with the notion of landscape of care notably highlight a shift in the topology of care taking place since the 1970s through deinstitutionalisation and the development of community care services, “one of the most profound social policy shifts in the history of Western welfare states” (Gleeson & Kearns, 2001, p. 1). This change implied the emergence of new sites of care, outside of traditional institutional settings (Milligan, 2009), due to both grassroot movements and the neo-liberal agenda of many states, which saw the possibility to save money while drawing more on unpaid work by families and volunteers. In what regard aging, there has been a shift towards ageing in place and community care, which “has brought into play new sites of care that are remote from traditional institutional settings” (Milligan, 2009, p. 21), requiring the co-production of care by families, volunteers, and professionals (Leyshon et al., 2019).

In summary, the concept of landscapes of care has the heuristic power to thematise complex sets of relationships, national and transnational regulations and policies, the geographical space, and the human and material arrangements in which older people live, in which they are cared for and about, and in which they may also care for and about others. However, this theorisation does not propose any systematic way of organising or analysing such complexity. Although social network analysis led to interesting mappings of care networks (see e.g. Fernández‑Peña et al., 2022), they focus on human beings and are thus far less inclusive than landscape of care approaches. Moreover, the concept of landscapes of care tends to oversee the activity of the people involved in the reproduction and transformation of the landscape. As we will see, sociocultural psychology, completed by the notion of boundary object, fills this gap.

### A Sociocultural Approach and Boundary Objects

Sociocultural psychology considers the mutual making of the persons and their social and cultural environment, and their changing relationship over time (Cole, 1996; Valsiner, 1998, 2000). It focuses, in various degrees, on people’s mediated activities and active meaning or sense-making, and assumes historicity, in both the sense that culture develops stabilised forms of experience through history (Boesch, 1991), and in the sense that people maintain continuity through their lives while developing and learning from experience (Zittoun et al., 2013). In this perspective, the specificity of development in older age can be characterised as follows: first, people with more age have a longer life experience, which implies more occasions to develop and learn; they may have learned from these experiences and developed “personal life philosophies” (Zittoun et al., 2013). Second, older people may have seen their historico-cultural environments change in substantial manner over a long period of time, which give them a unique perspective on the present (Hviid, 2020; Zittoun & Baucal, 2021). Third, older persons may be aware of the shorter time they have still to live, and experience a sense of finitude (Baars, 2017) that leads them to redefine the sense of their activities and to prioritise some of them. In this paper, however, we will not explore these developmental aspects further (but see for instance Zittoun, 2022). Rather, assuming that the environment is important for developmental processes, we will examine more closely the transactions between the person and their sociocultural environment. In order to describe these transactions, some researchers in sociocultural psychology have considered three interrelated scale of dynamics: *sociogenesis*, that is, the change in the social context — state, institutions, regulations, housings, etc.; *microgenesis*, or the daily, socially, and materially situated mediated interactions; and *ontogenesis* — the development of the person over time (Boesch, 2007; Duveen, 2001; Van der Veer & Valsiner, 1993; Zittoun & Baucal, 2021). To capture these different scales of dynamic changes and their interrelations, we propose to focus on *boundary objects* (Star & Griesemer, 1989) that can be apprehended along these three levels of analysis. The notion of “boundary object” designates objects that play a mediating role in people’s activities. They have a bridging function, allowing groups and persons “from different social worlds” (Star & Griesemer, 1989, p. 388), with different goals and perspectives, to coordinate their activities despite of the lack of consensus. In effect, boundary objects.

inhabit several intersecting worlds and satisfy the informational requirements of each of them [as they are] both plastic enough to adapt to local needs and the constraints of the several parties employing them, yet robust enough to maintain a common identity across sites. […] They have different meanings in different social worlds but their structure is common enough to more than one world to make them recognizable, a means of translation. (Star & Griesemer, 1989, p. 393)

Different theoretical traditions describe them as emerging between diverse communities of practice (Wenger, 1998), systems of activity (Engeström et al., 1995), or spheres of experience (Grossen et al., 2012) — all separated by a form of boundary. Boundaries can be defined as “sociocultural differences that give rise to discontinuities in interaction and action” (Akkerman & Bakker, 2011, p. 139), which are experienced as discontinuous by individuals or groups of people. Thus, boundary object might offer an opportunity to cross the boundaries between the different social worlds; thus, rather than obstacles that should be suppressed, boundaries and lack of consensus can be considered as potentials for learning, change, and development (Akkerman & Bakker, 2011; Engeström, 2001).

In summary, the notion of landscape of care enables us to approach older persons in the networks and spaces pertaining to different levels of analysis that support their lives and, more specifically, their everyday activities. Drawing on a sociocultural perspective, we propose to differentiate these levels of analysis and to pay special attention to the activities of specific actors and to the meaning they give to them. To articulate these different levels, we will examine how a specific object works or does not work as a boundary object between different groups of actors in the case of an evolving landscape of care.

## A Regional Case Study of an Evolving Landscape of Care

We will apply this theoretical framework to describe and analyse the case of an evolving landscape of care, a Swiss Canton engaged in a reform of its health, housing, and social policy, as part of the regional case study HomAge (Zittoun, 2019). We will focus in particular on flats with referees. This context provides an opportunity to apprehend human development through an articulation between sociogenesis, ontogenesis, and microgenesis. As the creation of these flats requires the participation of many social actors, both institutional and non-institutional, we ask whether these flats with referees work as boundary objects and fulfil a bridging function between the activities of those actors. In situations involving different groups and regarding specific aspects of the person’s support, will the flat with referees “satisfy the informational requirements of each of [the social worlds they inhabits]” (Star & Griesemer, 1989, p. 393)?

### Methodology

As part of HomAge, we combined desk research, interviews with experts and older persons, and visits of houses. The desk research focused on information regarding the cantonal situation in terms of housing possibilities and care services and arrangements for older people. We collected legal documents, minutes of political meetings, press releases, information communicated by media, and documents established by administrations and different types of organisations (e.g., information leaflet for older people and/or their relatives, business review). We conducted 110 semi-structured interviews, sometimes with several people at the time. We met 109 people in total. We saw some people several time in order to follow the evolution of the situation. Participants included politicians (3), employees of the cantonal and communal administration (5), directors and managers of fulltime institutions (6),the PMO of an important home care service (1), professionals health caretakers engaged in home care and in institutions (11), social workers and other professionals engaged in administrative support for older people (16), presidents and directors of organisations focused on informal caregivers and voluntary work (4), people engaged in the construction and management of apartments for elderly people (6) and caretakers working in such places (4), people engaged in the creation of intergenerational residents’ cooperatives and different forms of shared housing for elderly people (7), and older people living in flat with referees or interest in them (46). Interviews aimed at understanding participants’ perspectives on the landscape of care and of housing for older people, their representations of ageing, their goals, and their activities in the landscape. The interview questions were adapted to each person’s position in the landscape. We visited different places, often in relation with an interview: five buildings with flats with referees (three of these are recent, one is older but being renovated, one is under construction), a renovated building comprising two shared flats for elderly people, an intergenerational housing cooperative, a house comprising three flats for older women, and a nursing home also offering day care (Gfeller et al., 2021). As the HomAge project is collaborative, we also met the actors involved in order to present and discuss the results of our analyses (Akkerman et al., 2012; Gillespie et al., 2006). The data are presented here in an anonymised way.

## A Change in the Landscape of Care: Flats with Referees as Boundary Objects?

In this section, we start by providing some information about the transformations of the landscape of care in the Swiss Canton we study, and we present the introduction of flats with referees as one specific feature of this transformation.

What are the main goals of the new policy introduced in the Swiss Canton we study? The policy agenda, accepted unanimously by the parliament of this region, aimed at responding both to the wish expressed by a majority of older people (via a questionnaire) to stay at home as long as possible (Barbey et al., 2009), and to an economic goal to keep health expenditure bearable despite demographic changes. The program proposed, first, to reduce and limit the number of places available in medicalised, full-time nursing home; second, to create an important number of flats especially designed for ageing people, which we describe in more details below, and third, to deploy an ambitious network of care. The latter includes the organisation of an institution orienting people toward different offers, the creation of intermediary structures such as day-care centres and short stay units in nursing homes, the reinforcement of home care services, institutional support for caregivers and voluntary workers, and a better access to information for older people and their relatives. In other words, what we find here is an attempt, initiated at the political level, to modify the landscape of care for older people at a regional scale. This attempt is in line with the shift from mainly institutional care towards ageing at home and community care (Gleeson & Kearns, 2001; Milligan, 2009). Although it represents a more important involvement of the state in the domain of care for older people, this policy is in line with the Swiss neoliberal orientation (Schwiter et al., 2018) as it relies heavily on (unpaid) care by relatives and volunteers, individual responsibility, and the development of services provided by the private market. Moreover, older people and their families are expected to cover the cost of any non-medical care provision.

We propose a triple analysis of this transformation, across sociogenetic, microgenetic, and ontogenetic levels. We conducted an abductive analysis, with several movements between data production, work on data, and elaboration of the theoretical framework (Valsiner, 2014). Firstly, examining the data in a transversal manner, we collaboratively (Cornish et al., 2013) constructed an understanding of the flats, identified the main actors involved in it, their perspectives, as well as tensions between them, a phase in which the two other members of the HomAge project were also involved. We then organised the analysis into sociogenetic, microgenetic**,** and ontogenetic levels, selecting pieces of data that were relevant to this specific level and considering at each level to what extent the flats were indeed boundary objects, for whom, and in the context of which goals and activities. This part of the analysis was conducted by the two authors.

### Sociogenesis: Introducing Flats with Referees in the Regional Landscape

Flats with referees constitute an important part of the above mentioned planification elaborated by politicians. Their introduction constitutes a change at a sociogenetic level, as it aims at a lasting transformation of the landscape of care of the region. Flats with referees very soon appeared as potential boundary objects in this changing landscape of care, as different actors gravitated around them, conducted their activities in and through these flats, and thus, to some extent, articulated their diverse activities and trajectories with each other’s. A closer analysis of the actors involved, their activities and the situations in which their different activities met leads us to identify three different configurations, related to three different moments of the existence of flats with referees, in which flat with referees have a bridging function.

**Fig. 1:**
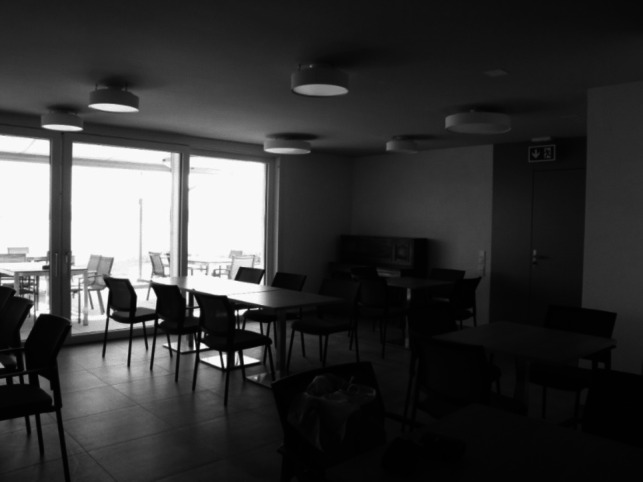
A common room (picture taken by F. Gfeller)

The first configuration corresponds to the planning and construction of these flats, which implies many actors with diverse professional backgrounds. The Canton has planned to build 2000 flats with referees in the next nine years. These flats are usually part of new or renovated buildings, owned by private companies, foundations, or public institutions. The building cost is borne by these actors who can nevertheless ask for financial support if the construction meets some criteria defined by the state. As part of the general policy aims at reducing the number of places in nursing homes, some of these flats are built by the foundations that own nursing homes so as to diversify their offer. Moreover, flats with referees have to follow certain urbanistic and architectural norms. Regarding urbanistic norms, the Canton has decided that these buildings should be constructed in each region or commune, so as to enable older citizens to remain in their own neighbourhood. Regarding architectural norms, the building and flats have to be accessible to people with reduced mobility: they should not have any threshold, any slippery grounds, and are equipped with walk-in showers, among others. They also need to fulfil a list of criteria regarding the immediate environment: they have to be located close to public transport, to shops, and cannot be situated on streets with a too steep declivity — a challenge in a hilly and mountainous canton. The flats with referees usually have two or three rooms; each building also includes a community room (see Fig. 1), and in some cases, large corridors that allow interpersonal meetings.

Hence, flats with referees become boundary objects in a first configuration, as they are located at the boundary of the activities of politicians, members of the public administration in charge of health and of housing (the two sectors who actively promote and control the construction and labelling process of flats with referees), promoters of the buildings and foundations leading nursing homes, diverse professionals with expertise in material arrangements for older people such as occupational therapists and suppliers of material for the construction: these various stakeholders have to coordinate to achieve their different goals (transforming the regional landscape of care, developing a financially interesting or at least sustainable housing supply, offering services for older people, selling kitchens, and so on). This may not go without tensions, difficulties and surprises.

The second configuration corresponds to the moment when older people decide whether they shall move to a new flat. Indeed, flats with referees aim to provide an intermediary solution between staying at home and moving to a nursing house; thus, they become a boundary object regarding older people’s decision about their place of living. From the policy makers’ point of view, the flats should offer one form of non-institutionalised (or faintly institutionalised) “ageing in place” and fit people’s wishes to stay at home (Barbey et al., 2009). In that sense, flats with referees become boundary objects between older people, as they respond to their wish to stay “at home,” understood here in the sense of “not moving to the nursing home” and living in one’s own private apartment, and policy makers, as flats with referees meet both to the politician’s goals of providing an economically sustainable response to demographic change and to their understanding of older people’s wish to age in place. However,”ageing in place” might have different meanings for different people (Forsyth & Molinsky, 2021). A paradox might notably appear when, from the perspective of the ageing person, moving to a new flat with referees is perceived as moving into another flat and thus as opposed to staying at one’s home. In this case, leaving their home is central in the person’s experience and this perspective contrasts with the understanding of flats with referees as a form of ageing in place.

The third configuration corresponds to the moment when older people moved to these flats and start living there. In this phase, flats with referees also work as a boundary object between tenants, their relatives, building owners, and professional caretakers intervening with the tenants, as it allows all of them to fulfil and articulate their activities, thus again playing a bridging function. Indeed, these flats aim at preventing older persons’ social isolation (Pitaud, 2010) through two measures. First, community rooms are meant to facilitate meetings and activities between the inhabitants. Second, a part-time professional, called “referee” and trained as social worker or nurse/care assistant, is responsible for visiting the tenants on a weekly or two-week basis. Referees are expected to have a little chat with the person, to check that everything is fine, and if not to arrange for assistance to be provided. They are also expected to organise group activities and to favour good neighbourhood relations among the tenants. These visits and activities are not compulsory for the tenants, but are included in the rental price. If tenants need additional homecare services — nurses, food delivery, etc. — they have to organise it themselves and pay for them (these are at times supported by health or social insurances).^2^ In this third configuration, the flats fulfil a function of boundary objects, as they offer to older people possible links with persons or agencies liable to provide support or care in situations where specific needs start to emerge and threaten the older person’s well-being and/or security.

Thus, at this sociogenetic level, flats with referees seem to accomplish a bridging function between different social worlds, which, according to Star and Griesemer (1989), is a characteristic of boundary objects. Indeed, on the one hand, soon after the project of flats with referees was introduced in the region, different promoters engaged willingly in the construction of such flats. On the other hand, once available on the market, the flats themselves were very quickly rented by older persons. These two observations seem to indicate that the project of flats with referees enables the coordination of different actors — notably the Canton, the promoters, and (at least) a part of the older population. However, we also observed situations in which the flats with referees created tensions more difficult to handle, and in which at least two actors or groups had difficulties cooperate.

### Microgenesis: Tensions Around Naming, Services Provided, and Aesthetics 

These tensions, observable at a microgenetic level, are among others related to naming, services, and aesthetics aspects of flats with referees.

We identify a first tension regarding the naming of flats with referees, that we characterise as a lack of common designation of the object. The state created an official quality label awarded to flats that meet all the criteria that turn a housing for older persons into flats with referees. This label is delivered by the public health office after a procedure involving examinations of the project, its construction, and visits to the finished building. Other buildings and flats targeted at an older population also exist in the Canton, but they do not receive the label because they meet only part of these standards; they do not have very clear specificities and some partly or even completely overlaps the standards of flats with referees. In this context, the creation of a label might be understood as an attempt to create some robustness and stability for the new object, which is an important characteristic of boundary objects (Star & Griesemer, 1989). Currently, 325 labelled flats with referees exist in the region, in seventeen different buildings. Several other buildings are under construction. However, the label is not really known neither by the population nor by professional caretakers. There is also some confusion about what the name covers and about the specificities of these various housing modes. Moreover, the term “flats with referees” is not commonly used, neither by health professionals nor by social workers, and laypersons use the terms “adapted,” “secured,” or “protected” flats, at times designating labelled flats, other times, not. Even the webpage of the Canton provides a list called “flats for older people” without any other indication. In other words, the creation of a label, not yet known and overlapping other names, testifies to the emerging and ambivalent status of these flats. Thus, despite the Canton’s will to create some robustness via labelling, there is an important uncertainty regarding what term is used for what type of housing, compromising the construction of a shared understanding of the meaning of “flats with referees.” In this regard, “flats with referees,” as boundary objects, do not completely achieve their function of bridge between various communities and persons, as they do not “satisfy the informational requirements” (Star & Griesemer, 1989, p. 393) of the population.

A second tension regards the services a flat with referees is supposed to provide to its inhabitants. On the one hand, like a nursing home, flats with referees are designed for older people (and younger people with reduced mobility), and thus follow a list of state-defined norms, and they have to offer specific services. Their construction is based on the assumption that older people may have specific needs, but that these needs do not necessarily limit their autonomy so severely that they need to move to a nursing home. For this very reason, flats with referees do not offer any medical care. In addition, according to the Canton’s recommendation, referees should not be medical staff or nurses, but social workers or other professionals used to work with older persons or trained to do so. However, there are uncertainties about what services are offered or can be expected in these flats. For instance, several misunderstandings were reported by referees regarding their presence in the building; tenants themselves or their relatives thought that the referees were present and available 24 h a day and every day, while their presence was limited to 3 days a week.

These misunderstandings, which reveal difficulties in the communication between different actors, were meanwhile clarified in the cases that were reported to us. This clarification illustrates the coordination work that takes place around boundary objects, a work that is necessary for the divergences in perspectives to become opportunities for learning (Akkerman & Bakker, 2011; Gfeller et al., 2023; Ros & Grossen, 2020). Part of this confusion may be due to the fact that sometimes the foundations managing nursing homes are those that build flats with referees in the close neighbourhood of their nursing home. Doing so, they diversify their offer and compensate for the loss of income due to the closure of places in the nursing home. They can also propose synergies between two modes of housing: people who live in the flats with referees can for instance have lunch in the nursing home. However, tenants of flats with referees are supposed to live a more or less independent and autonomous life, like in any “usual” flat on the housing market. As a result, and because of the proximity of the nursing home, they do not really know what to expect, what will be offered to them, or what is their margin of freedom; moreover, referees themselves are not always sure of the scope and limits of their own offer.

However, beyond possible misunderstandings regarding what is provided, it appears that several older people would prefer to have their own flat (as it is the case in flats with referees) but with the permanent presence of medical staff (as it is the case in a nursing home). In the case of uncertainty, the tension might disappear through discussion and clarification; in other cases, the clarification brings older people to renounce moving to these flats. While tensions appear as opportunities for learning in both cases (Akkerman & Bakker, 2011), the flats with referees respond to the needs of the different actors so as to allow them to fulfil their activities only in the first case.

One situation illustrates the microgenetic work of definition of the function of these flats and the services they should provide. One of the first buildings of flats with referees in the Canton was constructed about 5 years ago and is managed by a foundation also managing several nursing homes and day care services.^3^ The flats, although in a separated building, are very close to a nursing home. Tenants can benefit from services provided by the nursing home (implying however additional fees) such as meals, cleaning, or laundry service. The referee has been working in the nursing home for many years, and the director is the same person for both structures. At the open-door day, before moving in, a person asked whether it would be possible to bring her own carpet in the flat. The referee, used to the rules applied in the nursing home, answered it would not be allowed for security reasons. The administrative manager, however, corrected this information later on: as these are rented flats and thus private spaces, tenants are free to have carpets if they wish. Professionals and managers can advise them not to have some for security reasons, however it cannot be forbidden.

In this case, we observe that different social dynamics and perspectives collide around flats with referees: the rules of a nursing home vs rules of the private housing market; social and health professionals, and tenants; social workers and managers. In this specific instance, the manager played the role of a translator able to clarify the situation. Hence, this aspect of the flats with referees could become shared at least between the administrative manager and the referee, which — we can imagine — allowed them to continue to cooperate around this boundary object.

A third tension appears around the aesthetics of the flats and their functionality — another aspect of their status of boundary object. Different groups of people have a say on the appearance and technical specifications of the flats: architects, social and health workers, and tenants; each may be critical regarding each other’s priorities. Three examples can be mentioned.

**Fig. 2:**
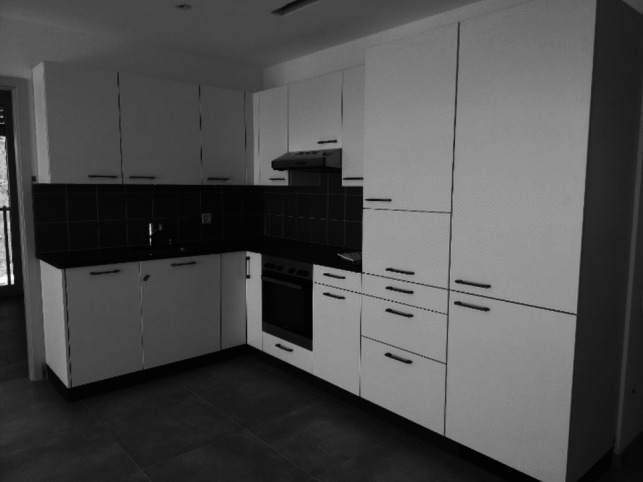
The height of kitchen cabinets (picture taken by F. Gfeller)

In the first case, a tension appeared between architects and promoters on one side and caretakers and tenants on the other side. Architects followed the complex regulations that constrain the construction of flats with referees. Despite of that, professional caretakers criticised the height of window handles and kitchen cabinets for functional reasons, notably for being too high for people in wheelchairs; several tenants also mentioned that they cannot use the upper part of kitchen cabinets because it is too high for them, even as they are standing (see Fig. 2).

Regarding the kitchen cabinets, architects and promotors justified this choice by the lack of alternatives as the space was limited; putting them lower would have reduced the work surface too much. It should also be said that the placement of handles and kitchen cabinets follows widely shared standards in house constructions nowadays and are also constrained by the material available on the market. Caretakers, who discovered these handles and cabinets once the flats were ready, expressed their wish to be consulted during the design and building of the flats. The initiators of the project however objected that the conception and building was already a fairly complicated process, as it implies many actors who try to comply with many (sometimes contradicting) regulations. Adding another point of view was perceived as adding more complexity with the risk to delaying the progress of the construction — a threat largely related to financial issues and to the conditions of the bank loans.

In the second case, tensions arose between the foundation initiating a housing project and the architects, the latter being criticised by the former as giving too much space to aesthetics and design. The discussion concerns the location of the balconies. The architect placed them on three sides of the building so that they were not too close to each other and that they are not in line with each other. According to him, this arrangement resulted in a harmonious facade. However, the initiators thought that from the users’ point of view, the balconies were not always placed in the best way regarding the setting of the flat (from inside) and the orientation (towards the village rather than towards the lake).

In the third case, the disagreement, which was reported by the initiator of the project, took place between the tenants and the architect. The architects chose to cover the kitchen floor with waxed concrete. The tenants, who had mostly lived in traditional flats or countryside houses, considered the floor as not functional as it quickly gets spots that are impossible to eliminate, and not aesthetic as the choice of a fashionable material gave them the impression that the building was unfinished. Here, diverging points of view concerning aesthetics and functionality collide.

In these three examples, the status of flats with referees as boundary objects is put at stake as no solution meeting the different groups’ interests and wishes was found. And, of course, the solution is all the more difficult to find as the disagreement concerns material aspects and appears when the building is already constructed. Borrowing Star and Griesemer’s (1989) words, we could say that the objects are not plastic enough anymore. This illustrates the importance of integrating different points of view at the right stage of the elaboration of the object.

### Ontogenesis: When Flats with Referees Meet Older People’s Life Trajectory

Paying attention to the ontogenetic level allows integrating older people’s life trajectory and to better understand how they encounter these types of flats, face the difficulties, and develop hopes and opportunities. As we have seen above, indeed, policy makers conceive of these flats with referees as “ageing in place” and “intermediary” forms of housing, while, from the viewpoint of those concerned, they require to move into another flat and to face a transition which might threaten their sense of continuity and integrity (Grossen et al., 2021). It shows how important it is to understand how people anticipate and make sense of their move in order to evaluate whether the flat responds to the person’s requirements and plays the bridging function attached to boundary objects. Two examples illustrate a matching between the person’s trajectory and the facilities provided by a flat with referees.

**Fig. 3:**
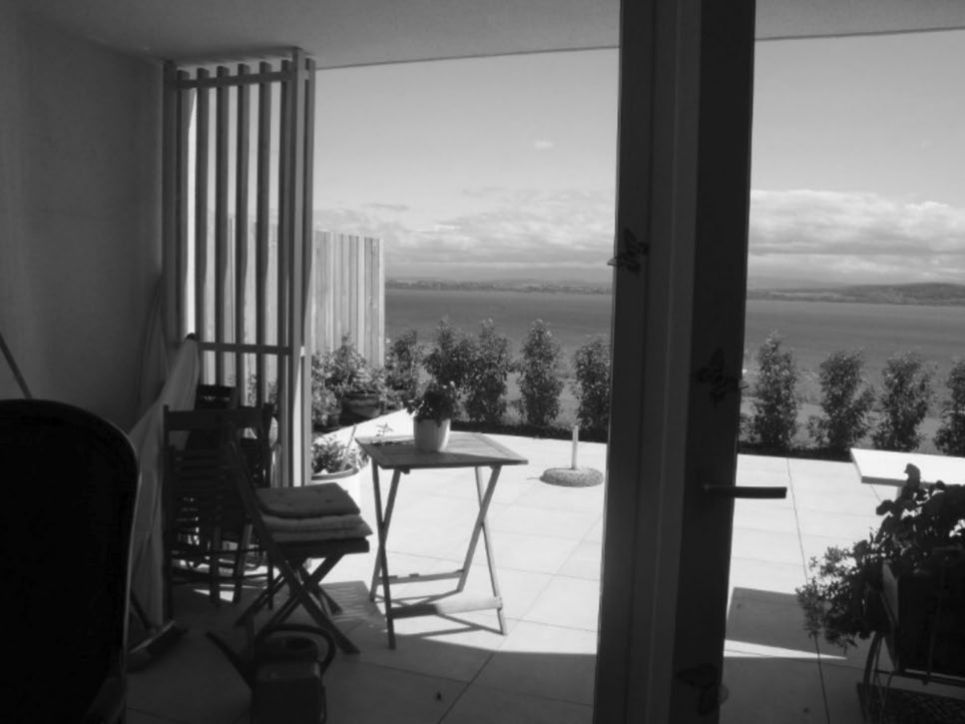
The view from the apartment (picture taken by F. Gfeller)

Firstly, the importance of keeping a certain continuity between their former housing arrangement and their new home appears, indeed, to be important for many older people (Cooney, 2012). In our past work, we have shown that the objects that older people can take with them from their home to the nursing home can support this sense of continuity (Grossen et al., 2021; see also Pazhoothundathil & Bailey, 2020). In the present study, we found another resource that people use to keep a sense of continuity: maintaining a contact with some aspect of the physical landscape, for instance the view on a specific mountain or on a lake — an aspect that appeared in the discourse of many older people who have to consider moving to another house. If the flat with referees meets this requirement (see Fig. 3), moving is facilitated. Thus, the location of the building might make it possible for the flats to play the role of a boundary object between the initiators of the project and the tenants.

For the former, the location meets financial and legal requirements, while they might be sensitive to the criteria of offering a beautiful view as it might help them to rent the flats, while for the latter, it enables the continuity of a contact with a specific geographic element. In this sense, people are literally “ageing in (their) place:” they change house, but feel that they keep their extended sphere of experience.

Secondly, the flats might respond to certain wishes regarding with whom one would like to share time and space. Moving into a flat with referees also implies changing neighbourhood and dealing with uncertainty about neighbourly relations. Buildings constructed in rural zones provide an opportunity to know, at least partly, who the other tenants will be. In one of the buildings we studied, several older women were looking forward, before they moved, to living in the same building as long-time friends. They spoke about it together, encouraged each other to move, talked about what they would do together once they would live there and shared tips about the organisation of the move. Most of them were living alone. Hence, although people move away from their house with the risk of losing part of their social network, or reinforce their social network by moving closer to kin resources (Badawy et al., 2019), we observed a different situation: people moved in the flats with referees with their social network, thus recreating and invigorating vital spheres of experience in this new building.

These opportunities and hopes are tidily linked to their past (for example, the relations they developed over time) and their future (how they imagine the next months and years) and are made possible as the building is constructed in a small village where people know each other, sometimes since school time. In this situation, the flats seem to fulfil their function of boundary objects as they respond both to the future tenants wishes — living in the same building as friends, and sharing activities with them — and to the objectives of the policy makers who imagined them also as a prevention against social isolation related to ageing.

## Discussion

We drew on the literature on landscapes of care to complement a sociocultural psychological approach to human development, focusing on the introduction of a new housing mode for older people in a region. The concept of landscape of care enabled us to capture sociogenetic transformations at the scale of a region. These echo the wider trend of “ageing in place,” responding both to humanistic calls and to neoliberal policies. At this sociogenetic level, the flats with referees designed by the Canton meet the needs and goals of several groups of people, older people who become tenants of these flats but also different types of stakeholders. We showed that considering them as boundary objects provides a theoretical explanation for the success of these flats among the different social groups involved in them at different stages. The concept thus renders visible the necessity of such collaboration. However, our sensitivity as sociocultural psychologists brings us to pay attention to people's engagements (Hviid, 2020) and “what matters” to them (Edwards, 2011; Gfeller et al., 2023), essential for people’s development and for possible interpersonal and intergroup collaborations. This invited us to examine the plurality of perspectives and goals among the different actors at a microgenetic level. There, we could identify tensions involving various groups of actors around the flats. In some cases, these could be resolved; the flats played their bridging function as boundary objects, and it seems that divergences were opportunities for learning (Engeström, 2001). In other situations, and notably when the object of the tension was a materialised aspect of the flat (handles, balconies, etc.), some aspect of the flat did not respond to some people’s goals, wishes, or perspectives. In these cases, we argue that the status of boundary object of the flats is at stake, as certain persons might stop their engagement with the flats as those did not respond to their requirements (Star & Griesemer, 1989) and the differences were not overcome (Akkerman & Bakker, 2011).

Finally, we also briefly showed how the older person’s trajectory — the ontogenetic level — plays a role in the way a person encounters a shared object such as a flat with referees. It invites to pay a close attention to biographical implications of the transition towards a new flat, a key moment according to studies on life trajectory, which involves meaning-making processes and can be supported by various resources (Zittoun, 2006, 2007). Note here the interest in considering the landscape — in the spatial, geographical, aesthetic, and symbolic sense — when considering the development of older people.

To pinpoint the multiple transactions across these dynamics and temporality, the concept of boundary object thus interestingly makes visible tensions of the field, and shows how, and in which conditions, these tensions lead to the cooperation of different actors (architects, politicians, older persons) and thus development, and when these failed to do so. Our observations thus enrich the concept of boundary object (Star & Griesemer, 1989); around a material, social and symbolic object that is a new mode of housing, we argue, it is not enough to consider the interests of different social groups: here, one has also to pay attention to the different temporalities involved — that of a building company is not the same than that of an older person moving flat, and the concrete and symbolic implications are very different.

The ageing of the population is a global problem and an international priority. It is of foremost importance for social sciences and psychology to work toward complex and integrative conceptual tools that enable us to read and participate in the transformation of our societies.

The study presented here contributed to this aim by providing a renewed theoretical frame to identify and analyse the development of older persons from a sociocultural perspective. The idea of the sociocultural world is concretely translated in that of landscape of care, and the articulation of sociogenetic, microgenetic, and ontogenetic dynamics gives us access to the many processes by which transforming environments participate to the development of older persons, and how these may, in turn, play a role in the evolution of these environments, or landscapes of care. Future analysis might articulate more specifically these theoretical constructs with the specificities of older peoples’ development, among which their longer life experience, their unique perspective on the present through the experiences of significant changes in the historico-cultural environment, and their sense of finitude.

## Data Availability

The datasets generated and analysed during the current study are not publicly available as individual privacy would be compromised. Metadata and grey literature will be available at the end of the HomAge project on reasonable request.

## Footnotes

1 “Housing modes and development in older age,” funded by the Swiss National Science Foundation, 2019–2023, 10001C_182401, directed by Prof. Tania Zittoun.

2 Note that if these flats with referees are mainly thought for older people, they can also welcome younger people with special needs, and some local actors thus also emphasize the intergenerational nature of these buildings.

3 Based on an interview with the director of these flats, of the near-standing nursing home and of another structure for older people with specific psycho-social needs, and with the administrative manager of the foundation.
